# The morphology of a smartphonopathic hand – smartphone use and the median nerve cross-sectional area

**DOI:** 10.12669/pjms.41.1.9958

**Published:** 2025-01

**Authors:** Syed Wajahat Hasib, Ambreen Usmani, Syed Faraz Anwar, Asra Mumtaz

**Affiliations:** 1 Syed Wajahat Hasib, M.Phil. (Anatomy) Bahria University Health Sciences Campus (BUHSC), Karachi, Pakistan; 2 Ambreen Usmani, MBBS, MPhil (Anatomy), MCPS-HPE, PGD-Bioethics, PhD (Anatomy), FCPS (Anatomy) Jinnah Medical and Dental College, Karachi, Pakistan; 3 Syed Faraz Anwar, MBBS, FCPS (Surgery), FCPS (Ortho), OJT Bone Tumor (UK), PNS Shifa, Karachi, Pakistan; 4 Asra Mumtaz, Pharm. D, MPhil (Pharmacology) Karachi, Pakistan

**Keywords:** Hand, Median Nerve, Smartphone, Smartphone Addictions, Carpal Tunnel Syndrome

## Abstract

**Objective:**

To investigate and compare the median nerve-cross sectional area at the wrist region of the dominant and non-dominant hands of high- and low- smartphone users.

**Method:**

This descriptive cross-sectional study was based on 128 human subjects conducted at Bahria University Health Sciences Karachi Campus from January to June 2022. The sample size was calculated using the method of sample size for frequency in a population www.openepi.com which is an open-source calculator, version 3-SSPropor using the following equation: Sample size n = [DEFF*Np(1-p)]/[(d^2^/Z^2^1-α/2*(N-1)+p*(1-P)]. The subjects were from MBBS, BDS, DPT, and Dental House Officers. They were grouped into two categories: high-smartphone users and low-smartphone users, based on the smartphone addiction scale (SAS). Ultrasound was carried out on each subject’s wrist regions at the carpal tunnel level. The readings were compared between the dominant and non-dominant hands of each individual.

**Results:**

In this descriptive cross-sectional study on 128 subjects, the difference between the median nerve cross-sectional areas (MN-CSAs) of the dominant and non-dominant hand of the high-smartphone group was highly significant (*p*=0.007). The difference between the MN-CSAs of the dominant and non-dominant hand of the low-smartphone group was significant too (*p*=0.0103).

**Conclusion:**

Smartphone overuse resulted in an enlarged median nerve, especially in the dominant hand.

## INTRODUCTION

Over the decades, numerous inventions have emerged, improving and complicating lives. Perhaps, out of all those, the most ingenious one is the devise of handheld devices, particularly smartphones. Today’s smartphones resemble minicomputers.^1^ A smartphone can be defined as a hybrid between a computer and a telephone.^2^ Among handheld devices such as pagers, laptops, smartwatches, digital cameras, and smartphones, the latter have taken the world by storm and their usage has boomed in the last decade or so. In 2013 it was found that there were nearly as numerous mobile subscriptions as individuals in the world.^2^

The number of smartphone users by the year 2020 had reached 3.5 billion globally, 9.3% higher than what it was in 2019.^3^ Commonly, the posture when operating a smartphone involves clutching it single or with both hands below eye level, looking down at it, and using the thumb to glide and tap across the screen of the smartphone.^4^ The limited number of studies that have been conducted have reported that continuous finger movements, unnatural wrist position, and forceful exertion such as applying excessive force while typing or scrolling can further increase tension in the wrist and might lead to carpal tunnel syndrome (CTS) and deformation of the median nerve after operating a smartphone for 30 minutes.^5^ Symptoms of CTS commonly include limited hand movement along with burning pain in the part of the forearm and hand innervated by the median nerve.^6^

The median nerve is a mixed nerve that arises from the brachial plexus, a collection of nerves from C6 to T1 that supplies the upper limb.^7^ After traversing the forearm, the median nerve, positioned centrally, heads for the carpal tunnel.^8^ It enters the carpal tunnel with the nine flexor tendons related to the digits (fingers) and pollicis (thumb). Despite being communicable on its proximal and distal ends, the carpal tunnel exerts a specific pressure which is referred to as the carpal tunnel pressure, the normal value of which is between 2-10 mmHg.^9^

CTS can occur due to many factors. These can be classified into modifiable and non-modifiable risk factors. The former comprises settings that require continuous use of the hands and wrists. This includes individuals working in factories, construction sites, and offices, who constantly use a keyboard, type, or operate machinery. Unergonomic postures of the wrists, obesity, and smoking are also modifiable risk factors. Some of the non-modifiable risk factors include age, gender, genetics, and wrist injury.^9^ The median nerve can suffer ischemic injury due to compression force exerted by increased intrafunicular pressure that results in capillary trauma. When the intracarpal pressure reaches constant levels between 20-30 mm Hg, it becomes damaging to the median nerve.^10^

The narrowest diameter of the carpal tunnel, where the nerve morphology is found to be altered in cases of carpal tunnel syndrome, is about 2cm from its proximal edge.^11^ Ultrasound has been one of the gold standards for diagnosing CTS as it can detect thickening of the median nerve before the carpal tunnel, flattening of the nerve within the tunnel, and bowing of the flexor retinaculum, all diagnostic of CTS. Research has stated a cross-sectional area of 9 mm^2^ or more to be 87.3% sensitive and 83.3% specific for CTS.^7^

Literature has provided evidence of multiple adverse effects of smartphone overuse, but it is scarce on the adverse effects of smartphone overuse on the hand and its functionality. There’s no text available regarding the median nerve cross-sectional area being affected by smartphone usage in the respective country. Hence, the study aimed to investigate the relationship between smartphone overuse and the median nerve cross-section (MN-CSA) in medical university students.

## METHODS

This was a descriptive cross-sectional study based on 128 human subjects. The sample size was calculated using the method of sample size for frequency in a population www.openepi.com which is an open-source calculator, version 3-SSPropor using the following equation: Sample size n = [DEFF*Np(1-p)]/[(d^2^/Z^2^1-α/2*(N-1)+p*(1-P)]. The sample technique was purposive. The period of this study was from January to June 2022. It was conducted at the Ultrasound and X-Ray departments of a public hospital in Karachi. The materials used to conduct this study included a questionnaire, a smartphone addiction scale (SAS), consent forms in English and Urdu, and a diagnostic ultrasound system (Toshiba Aplio 500 model TUS-A500).

### Ethical Approval:

The ethical approval was granted by the Ethical Review Committee (ERC) of Bahria University of Health Sciences Campus Karachi (BUHSCK) bearing the approval number ERC 87/2021 dated 21^st^ December 2021.

The current study collected data through a self-administered questionnaire. The questionnaire comprised two parts. The first part collected the demographic details, past medical history, smartphone-related details, presence of any habits or hobbies (such as clicking the top of a ballpen, playing musical instruments, or other), exercise status, and usage of a laptop. The second part consisted of the smartphone addiction scale (SAS)^12^,^13^, a 33-item-based questionnaire. The distribution of questionnaires was to students of different medical disciplines and dental house officers.

After disseminating the consent forms which briefed the individuals about the research and the following radiological evaluation, the two-part questionnaire was distributed. The measurements of the MN-CSA were noted on a separate sheet. Inclusion criteria included students and dental house officers of every gender at BUHSCK between the ages of 17-25.

**Fig.1:**
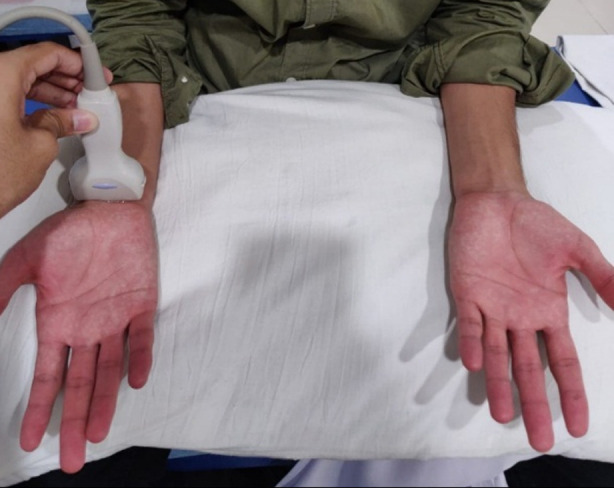
A subject undergoing ultrasound of right wrist at the distal wrist crease level (carpal tunnel inlet).

All those individuals were excluded who had a history of trauma or fracture of the upper limbs and individuals with any sort of neuropathy. Ultrasounds of both wrists were done by a clinician blinded to the SAS scores. A diagnostic ultrasound system (Toshiba Aplio 500 model TUS-A500) was used, and ultrasound was carried out with a linear array probe (13 MHz). Individuals were seated with a pillow over their legs, their arms (semi-flexed at around 90°) resting on the pillow, and their hands completely supinated (Fig.1). The transducer was placed axially at the carpal tunnel inlet denoted by the distal wrist crease on the anterior surface of the forearm.^14^

**Fig.2:**
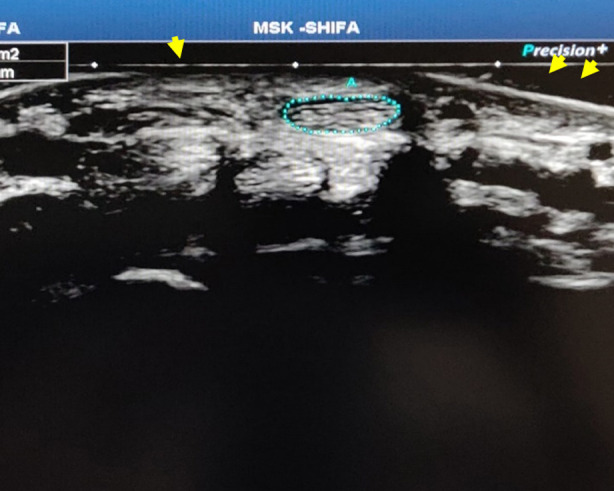
Flexor retinaculum (yellow arrows). The median nerve has been outlined/manually traced (blue dotted line).

Minimal pressure was applied by the transducer on the wrist. The median nerve was identified and confirmed by its superficial position just beneath the flexor retinaculum, its honeycomb appearance, and its stationary diameter upon moving the digits and pollicis (thumb) which identified the flexors of the hand as well and aided in differentiating them from the median nerve. The image was then frozen, and the cross-sectional area (CSA) was measured with axial imaging and manual tracing method (Fig.2).^15^ The dominant hand was assessed first followed by the non-dominant hand.

### Statistical analysis:

The analysis was done using IBMM SPSS 22.0. The data was checked for normality by the Shapiro-Wilk test and was proven not to be normally distributed due to the small sample size. The difference between the two groups (high- or low- smartphone users, and hand-dominance) was statistically assessed and compared using the Wilcoxon signed-rank test. The association between the MN-CSA in the dominant hand and hobbies, exercise, or laptop use was assessed using the biserial correlation test. The p-value was set to ≤0.05.

## RESULTS

The present study was conducted on 128 subjects from Bahria University Health Sciences Karachi Campus. The subjects were from MBBS, BDS, DPT, and Dental House Officers. The participants were aged 17-25 years with the mean age being 20.55 ± 1.787. Of the 128 participants, 43 (33.6%) were males and 85 were females (66.4%). Only 4 (3.1%) participants were left-dominant handed and the remaining 124 (96.9%) were right-dominant handed. Out of the 128 subjects, 83 had a hobby that required the use of the hand or digits, 100 owned a laptop, and 40 students actively exercised at the time of the study. The median score of SAS of the 128 participants was 114.5, the lowest score being 53 and the highest being 185. Subjects having a score higher than 114.5 were considered as high-smartphone users and those having a lower score than 114.5 were considered as low-smartphone users. An equal number of participants scored higher and lower than 114.5, resulting in 64 people in both groups.

Of the 256 ultrasounds done (128 x 2), the mean MN-CSA in the dominant hand of the high-smartphone user group was 7.95 mm^2^ while in the non-dominant hand of this group, it was 7.34 mm^2^. The difference between the two was highly significant (*p*=0.007). In the low-smartphone user group, the mean MN-CSA in the dominant hand was 7.16 mm^2^ (lower than the high-smartphone group) and 6.64 mm^2^ (lower than the high-smartphone group) in the non-dominant hand, the difference between the two was also significant (*p*=0.0103) (Table-I). The MN-CSA of the dominant hand did not show any statistically significant correlation to hobbies, exercise, and laptop use (Table-II).

**Table-I:** Median nerve-cross sectional area of the dominant hand and non-dominant hand compared.

	Dominant Hand	Non-dominant Hand	p
High Smartphone Users			
MN-CSA (mm^2^)	7.95±1.855	7.34±1.896	0.007**
Low Smartphone Users			
MN-CSA (mm^2^)	7.16±1.921	6.64±1.495	0.0103*

p-value < 0.05 = statistically significant (*); <0.01 = highly statistically significant (**) Test applied = Wilcoxon matched-pairs signed-ranks test.

**Table-II:** Correlation between MN-CSA in the dominant hand and hobbies, laptop use, and exercise.

	MN-CSA in dominant hand of all subjects (n = 128)	

	r_pb_	P
Hobbies	-0.051	0.568
Laptop Users	0.010	0.909
Exercise	-0.148	0.096

p-value < 0.05 = statistically significant (*); <0.01 = highly statistically significant (**); Test applied = Point biserial correlation.

## DISCUSSION

The results of our study showed significantly increased MN-CSA in the dominant hands of both, the high-smartphone and low-smartphone user, groups when compared with their non-dominant hands. An increase in MNCSA, due to smartphone use as observed, can also lead to carpal tunnel syndrome (CTS). MN-CSA > 9 mm^2^ proximal to the carpal tunnel, >10.03 mm^2^ in the proximal carpal tunnel, or >10.5 mm^2^ at pisiform bone level, is considered to be abnormal.^16^ This increase in MN-CSA due to high smartphone usage in the dominant hand, or smartphone usage in general, possibly leading to CTS, is supported by few studies done on this matter.

One of the pioneer studies investigating the relationship between median nerve and smartphone usage was conducted in Turkey that reported considerably increased MN-CSAs in the dominant hands of the high-smartphone user group than in the non-dominant hands of the same group, thereby supporting the results of our study. They were also the first to mention the term “Hand Smartphonopathy” which referred to the adverse effects of excessive smartphone use on the hand and suggested this term be coined if more studies were to report similar clinical findings.^16^

Another study^17^ also reported significantly high MN-CSA in the high-smartphone users as compared to the low-smartphone users; One study^18^ observed that the CSA of the median nerve was higher in the participants typing on their phone single-handedly than the ones typing with two hands, hinting that rapid movement of digit and extensive thumb-tapping activity resulted in swelling of the median nerve; Similarly, another study^19^ results showed that using smartphone at an increasing rate adversely influenced the median nerve; The results of our study were also supported by a study^20^ done in Hong Kong which reported that intensive electronic device users also had significantly enlarged MN-CSA, flattening ratios, and perimeters.

Our study provided more material to the scarce existing literature on the relationship between smartphone usage and its effect on the median nerve cross-sectional area (MN-CSA) at the wrist region and its possible adverse effects on hand function. The current study is also only the fifth study to shed light on the word “hand smartphonopathy” to add it to the literature signifying the detrimental effects of smartphone overuse on the hand^16^,^21^-^23^ and in doing so it is only the first study to put forth the term “smartphonopathic hand”. It also added to the other well-known and studied adverse effects of smartphone overuse, such as depression^24^, insomnia and blurred vision^25^, alexithymia^26^, and chronic neck and upper back pain.^27^

Our study showed that MN-CSA indeed increased in smartphone users, more so in the high-smartphone group than the low-smartphone group. This increase in the MN-CSA could lead to carpal tunnel syndrome over a prolonged period as suggested by a few other past studies. Hence, clinicians should take notice of these findings and thoroughly investigate the pattern and extent of smartphone usage in patients presenting with complaints of altered hand function, tingling sensation, and atrophied thenar eminence, without any significant medical history.

### Strengths:

By far, the sample size in the current study is the largest for this topic of research. It is also the first to be conducted in the respective country. To the best of our knowledge, this is the first study that considered and tried to exclude other factors that might affect the relationship between increased MN-CSA in the wrist and smartphone usage.

### Limitations:

Despite the current study having the largest sample size compared to previous studies, it is still small. Dental house officers, although very few, participated in the current study which could have provided bias in the results due to their wrist movements concerning their profession.

### Recommendations:

Future studies need to be conducted on a larger sample size to strengthen smartphone overuse as one of the risk factors for CTS. To do that, confounding factors such as usage of other handheld devices, occupation, and sports activities, also need to be considered.

## CONCLUSION

This study concludes that smartphone overuse leads to an enlarged median nerve cross-sectional area, more so in the dominant hand than the non-dominant hand of smartphone users.

### Author’s Contribution:

**SWH:** Conceived the research idea, designed and set the parameters and assessment tools, collected non-clinical data, wrote and edited the manuscript, and is responsible for integrity of research.

**AU:** Is the main supervisor who supervised the research, reviewed, interpreted the results, and gave the final approval of the manuscript.

**SFA:** Is the clinical supervisor and assessed the participants and collected the data, clinically.

**AM:** Did the statistical analysis of the research and manuscript preparation.
